# Exposure risk of patients with chronic infectious wounds during the COVID-19 outbreak and its countermeasures

**DOI:** 10.1186/s13018-020-01976-0

**Published:** 2020-10-02

**Authors:** Haiying Zhou, Qianjun Jin, Hui Lu

**Affiliations:** Department of Orthopedics, The First Affiliated Hospital, College of Medicine, Zhejiang University, #79 Qingchun Road, Hangzhou, 310003 Zhejiang Province People’s Republic of China

**Keywords:** Novel coronavirus pneumonia, Chronic wounds, High-risk populations, Clinical characteristics, Prognostic factors, Management, Retrospective study

## Abstract

**Background:**

A large number of cases of pneumonia caused by novel β-coronavirus emerged in Hubei Province, China, at the end of 2019 and demonstrated great potential for transmission. At present, known independent risk factors include age, diabetes, and other chronic diseases, which may be similar to the patients with chronic wound; thus, we try to explore the clinical characteristics, prognostic factors, and management recommendation of patients with chronic infective wounds during the COVID-19 epidemic period.

**Methods:**

In this single-center, retrospective observational study, we included all cases with chronic infective wounds that came to our hospital between the full outbreak of the COVID-19 in China (January 23, 2020) and the latest date prior to posting (20 April 2020). Demographic data, comorbidities, laboratory and imaging findings, consultation history, and clinical outcomes (lesion cured, uncontrolled, amputated, etc. as of May 10, 2020) were collected for all individuals. Patients were subdivided into gangrene, traumatic infection, and other types of soft tissue infection wound (including bedsores, gout ruptures, stab wounds, and so on) according to the causes of wound, and their disease-related information were compared group by group.

**Results:**

Among the total 81 patients with chronic infective wounds, 60% were male, with a mean age of 60.8 years (SD 18.6), including 38 (47%) patients with traumatic infection, 29 (36%) gangrene cases, and 14 (17%) other soft tissue infection wounds. Common comorbidities are hypertension (32%), diabetes (32%), cardiovascular disease (24%), and kidney injury (12%), and the patients with gangrenes have the most comorbidities. As of May 10, 2020, there were 78 patients discharged, and their average stay time is 15.8 days (SD 14.2), while people still at the hospital is 39.7 days (SD 8.7) much longer than the discharged and also has more comorbidities. But there is no significant difference in the hospitalization time of three types of wounds. And fortunately, none of all the patients were infected by coronavirus.

**Conclusion:**

The majority of patients with chronic wounds are severely ill with high risk of infection and poor prognosis; therefore, management of patients with chronic wounds should be improved.

## Background

Coronavirus disease 2019 (COVID-19) caused by severe acute respiratory syndrome coronavirus 2 (SARS-CoV-2) infection was first detected in Wuhan, Hubei Province, China, then quickly spread throughout China and spread widely around the world, causing concern and alarm among people all over the world. As of 12:30 p.m. Beijing time on September 06, 2020, according to real-time data released by Johns Hopkins University, there were a cumulative total of 26,873,146 confirmed COVID-19 cases and 879,307 death, making COVID-19 a worldwide pandemic disease. SARS-CoV-2 has been proved to be highly contagious, for its real-time effective reproduction number in Italy can even reach 6.56 [1, 2]. When people are affected, it has diverse clinical manifestations and atypical symptoms, seriously threatening the life and health of the population [3, 4].

Studies have shown that SARS-CoV-2 is a kind of the realm RNA virus (subgenus sarbecovirus, β-genus, orthocoronavirinae subfamily) and is susceptible for human beings [5, 6]. However, due to its unique hardened protein shell, its propagation potential is vastly higher than that of MERS and SARS [7, 8], which has been shown to be transmitted by droplets and close contact; at the same time, fecal-oral and blood-borne routes are still being studied [9, 10]. Meanwhile, the general susceptibility of the population is so widespread that, if not controlled in time and effective, the rapidly increasing number of cases will increase the burden of medical system and eventually lead inevitably to a collapse of health care, but on the contrary, timely and effective identification and isolation of patients will significantly curb the spread of the disease [3, 11, 12]. Zhang et al., establishing a COVID-19 transmission model based on survey data collected in Shanghai and Wuhan, demonstrated that social distance policies alone were sufficient to control COVID-19 during outbreaks in China [13]. Unfortunately, in the early stages of the disease, the patient’s symptoms are mild and generally present as common influenza symptoms, with fever, dry cough, and fatigue being the main manifestations [3, 14, 15]; however, if the patient is not effectively treated and isolated, not only will the virus spread [16], but the disease will also progress to acute respiratory distress syndrome, respiratory failure, and even death [17].

Chronic wound is defined as a wound that has failed to achieve anatomical and functional integrity through orderly and timely repair within 3 months [18], and is prevalent in the population with the number of patients increasing year by year [19–21]. Besides, similar to COVID-19, the middle-aged population and diabetics are at high risk of chronic wounds [11, 19, 22]. Although wound non-healing is considered multifactorial in nature [18], infection and microbial implantation may be the single most likely and unavoidable cause of delayed wound healing [23, 24], and without timely and effective treatment, bacterial implantation in the body can lead to systemic infection or even life-threatening infection [25, 26]. At the same time, removing the dead tissue also plays an important role in wound healing, thereby regular debridement and surgical or medical treatment is necessary. While patients can self-treat their wounds with simple treatments such as disinfection and dressing changes, even so, studies have shown that self-treatment of wounds can still benefit from more professional, standardized outside supervision and assistance [27]. Therefore, going to the hospital is an outing that remains unavoidable during the epidemic of COVID-19 for patients with chronic infective wound, which may increase their risk of COVID-19 infection.

Up to now, there are few reports in the literature on the clinical characteristics of patients with chronic infective wounds during COVID-19, and its associated risk factors have yet to be investigated, which has important implications for the identification and diagnosis of COVID-19 among patients with chronic wound in the clinic. Therefore, this paper proposes to investigate the relationship between chronic wound and the risk of COVID-19 infection by reviewing their common clinical characteristics, hospitalization process, and prognosis, so as to provide clues and basis for physicians to identify COVID-19 patients during the clinical consultation. Besides, by analyzing the relevant factors affecting the prognosis of patients with infectious wounds, we are trying to provide recommendations for the implementation and selection of actual treatment, which may be helpful to improve clinical practice and prognosis of this kind of patients.

## Methods

This single-center, retrospective observational study was conducted at the First Affiliated Hospital, College of Medicine, Zhejiang University (Hangzhou, China), which is also the designated hospital for the treatment of novel coronavirus pneumonia in Zhejiang. We retrospectively analyzed all cases of chronic infectious lesions that came to our hospital between the time of the full outbreak of COVID-19 in China (23 January 2020) and the latest date prior to posting (20 April 2020). Information on age, comorbidities, medical history, laboratory tests, imaging findings, and clinical outcomes were collected, including laboratory confirmation of SARS-CoV-2 infection by the local health department. The patient’s survival status was confirmed on May 10, 2020.

We reviewed the clinical electronic medical records, visit records, laboratory findings, and radiological examinations of all visit patients with chronic infective wounds, and collected and corrected missing or uncertain parts of the record through direct communication with their attending physicians and family members. Afterward, based on the cause of their wound, we classified patients with chronic wounds as gangrene due to vascular occlusion, ulcers due to traumatic infection, and other types of soft tissue infection wound due to bedsores, gout ruptures, and stabbing. Risk factors for coronavirus were limited to advanced age (≥ 60 years), hypertension, diabetes, renal damage, and cardiovascular disease according to the available studies [3, 11, 28].

We collected data on the number of patients with chronic infective wound that attend our hospital between 23 January 2020 and 20 April 2020: age, sex, medical condition, other chronic medical histories (e.g., chronic heart disease, hypertension, cardiovascular disease), laboratory findings, treatment options, length of hospital stay, survival, etc. We described quantitative data by means and standard deviation (SD), categorical data by percentages, and used one-way ANOVA to analyze the differences in length of stay for different types of wound and a Chi-square test to analyze the differences in COVID-19 risk factors for patients with different types of wound, using SPSS 23.0 for all analyses.

Written informed consent was obtained from each patient for publication of this article and any accompanying images. Ethical approval was provided by the medical ethics committee of the First Affiliated Hospital, College of Medicine, Zhejiang University.

## Results

**Fig. 1:**
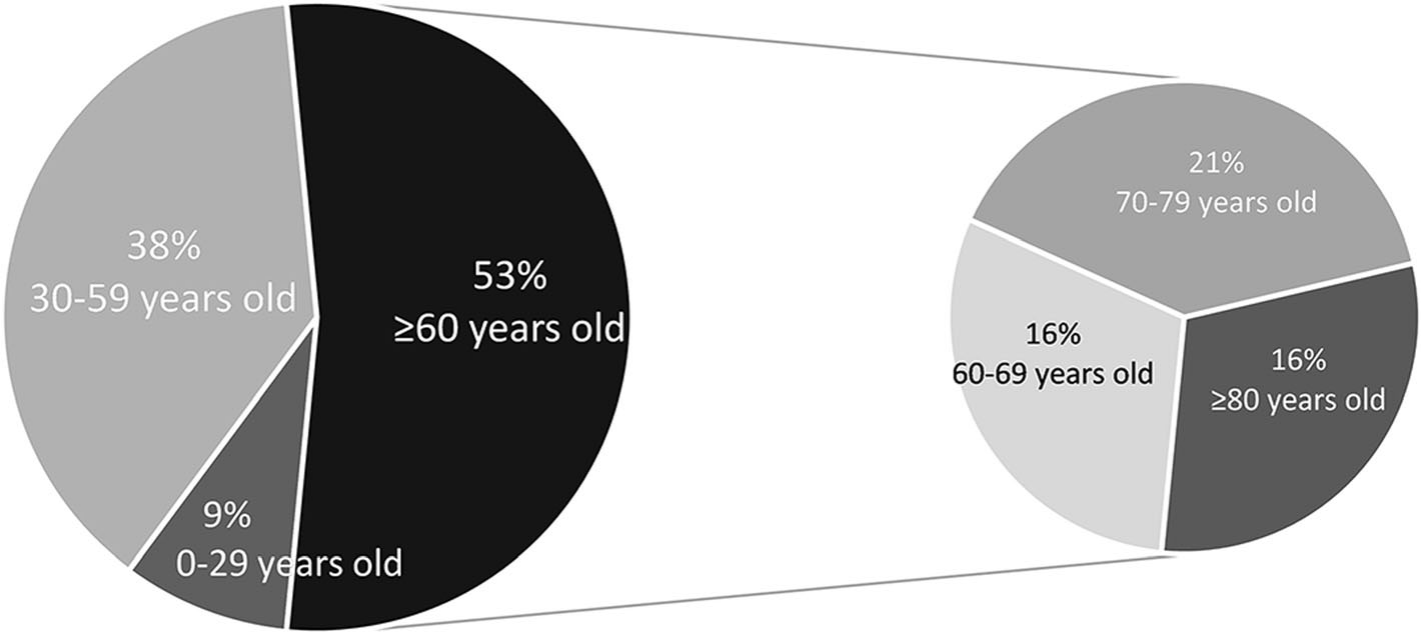
Proportion of patients of different age groups

**Fig. 2:**
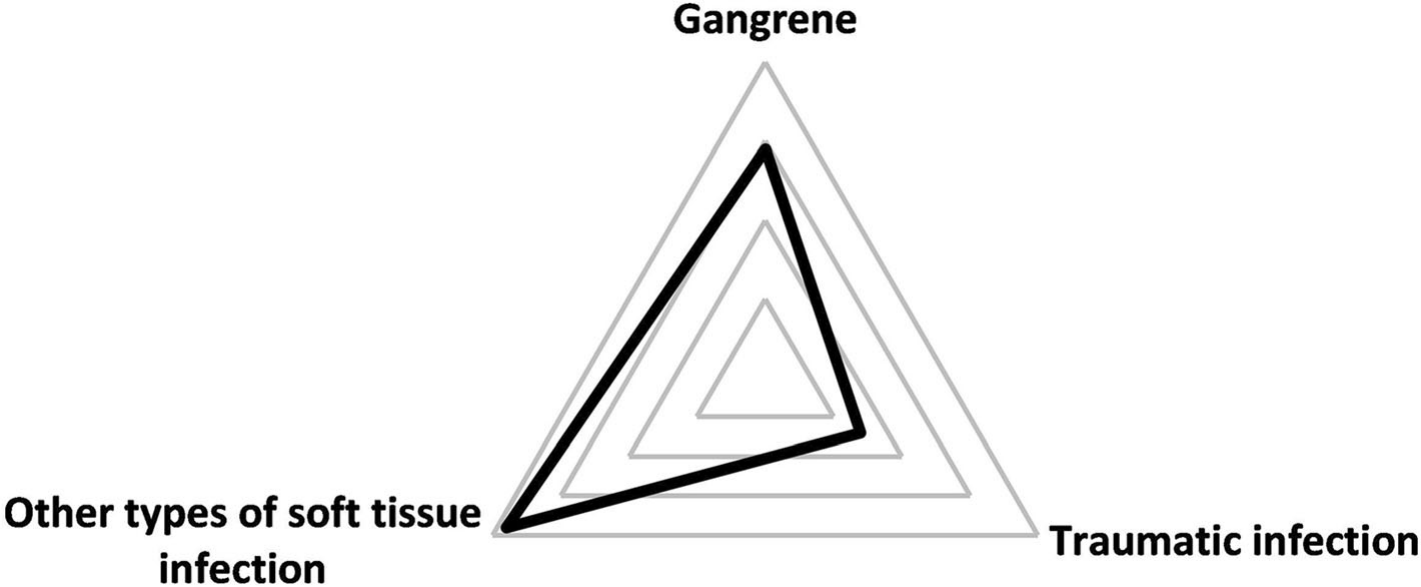
Number of patients of various types of wound

**Fig. 3:**
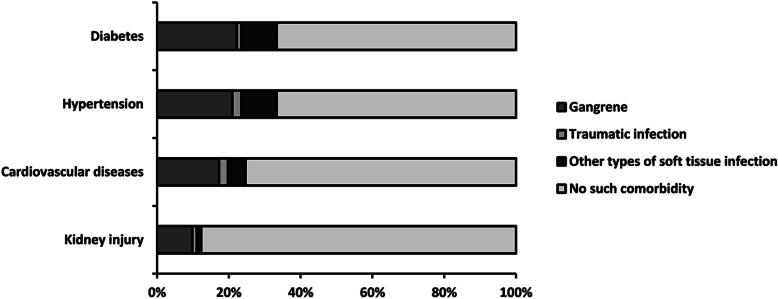
Distribution of patients with different basic diseases

By April 20, 2020, a total of 81 patients with chronic wounds came to our hospital for treatment, and Table 1 illustrates some of the main characteristics of those patients. It can be seen that the patients are predominantly elderly and male, which accounts for 60%, and the average age is 61.2 years (SD 19.1), as well as 53% of the total were over 60 years old (Fig. 1). Forty-seven percent of patients had other types of soft tissue infection wound, while the gangrenes due to vascular occlusion occupying 36% in total are the second (Fig. 2). Common comorbidities were diabetes (32%), hypertension (32%), and cardiovascular disease (24%) (Fig. 3). Fortunately, in our investigation, all patients were free of COVID-19 infection.

**Table 1:** Demographics and clinical characteristics of patients with diabetic foot

Characteristics		Total (***n*** = 81)
Age, years		60.8 (18.6)
Age range, years	0–29	7 (9%)
	30–59	31 (38%)
	60–69	13 (16%)
	70–79	17 (21%)
	≥ 80	13 (16%)
Sex	Male	49 (60%)
	Female	32 (40%)
Type of wound	Gangrene	29 (36%)
	Traumatic infection	14 (17%)
	Other types of soft tissue infection	38 (47%)
Comorbidity	Hypertension	27 (32%)
	Diabetes	27 (32%)
	Kidney injury	10 (12%)
	Cardiovascular diseases	20 (24%)
Length of hospital stay		16.7 (14.7)
Infected with COVID-19		0
Death		1 (1%)

Data are *n* (%) or mean (SD). One patient died of septic shock

**Fig. 4:**
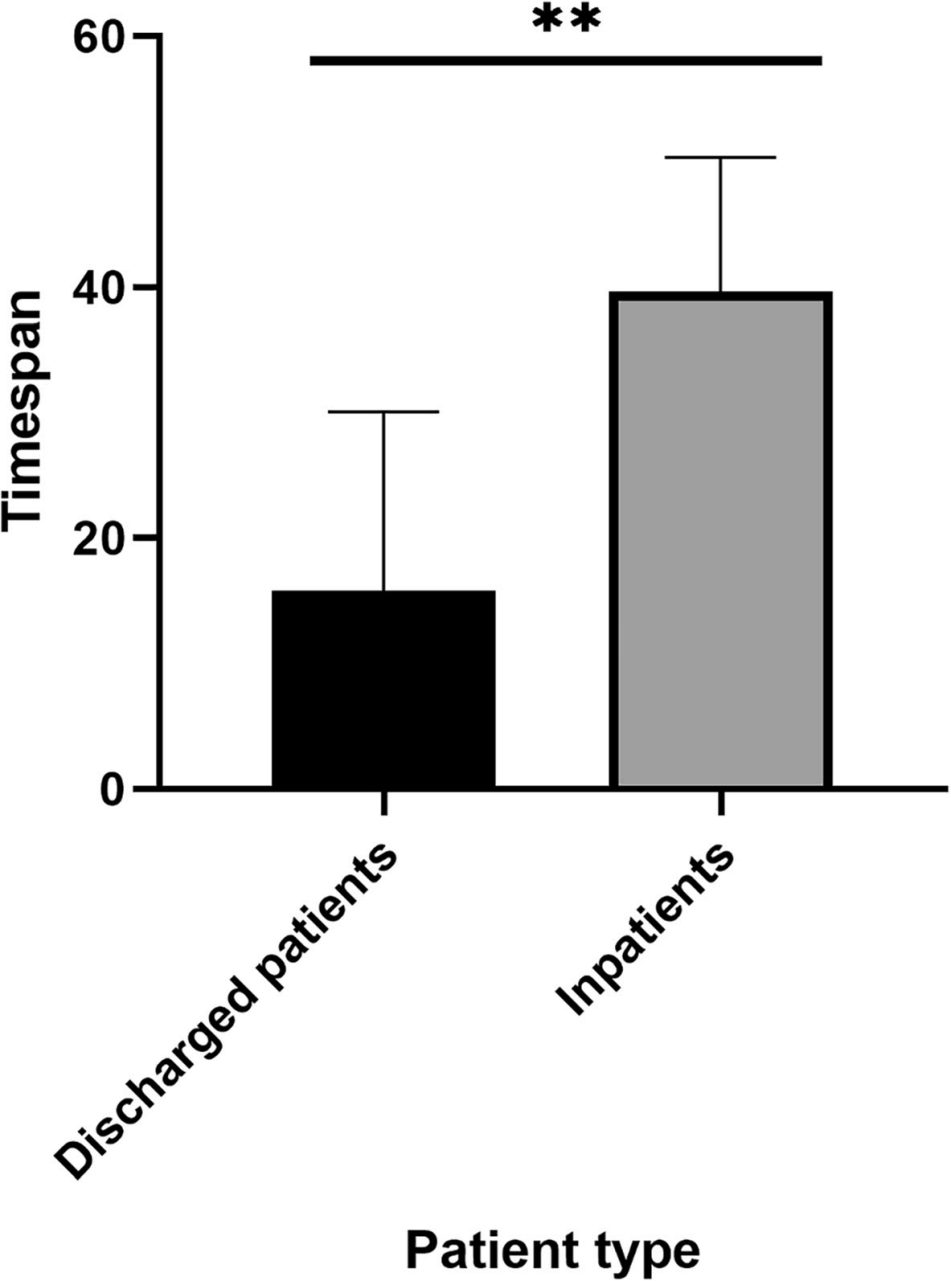
Comparison of the length of hospital stay between discharged patients and patients still in hospital. *n* = 81, *p* value > 0.05 not marked, *< 0.05, **< 0.01, ***< 0.005, ****< 0.001

**Fig. 5:**
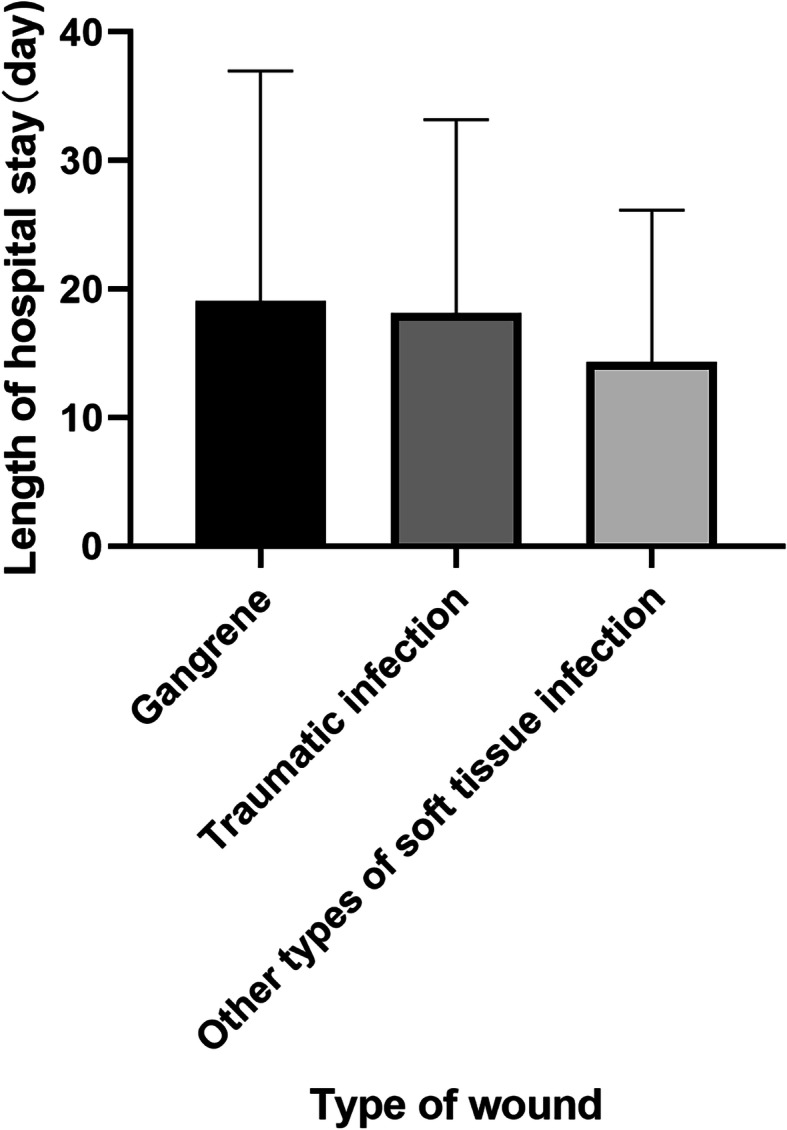
The difference of hospitalization time in different types of wound. *n* = 81, *p* value > 0.05 not marked, *< 0.05, **< 0.01, ***< 0.005, ****< 0.001

**Fig. 6:**
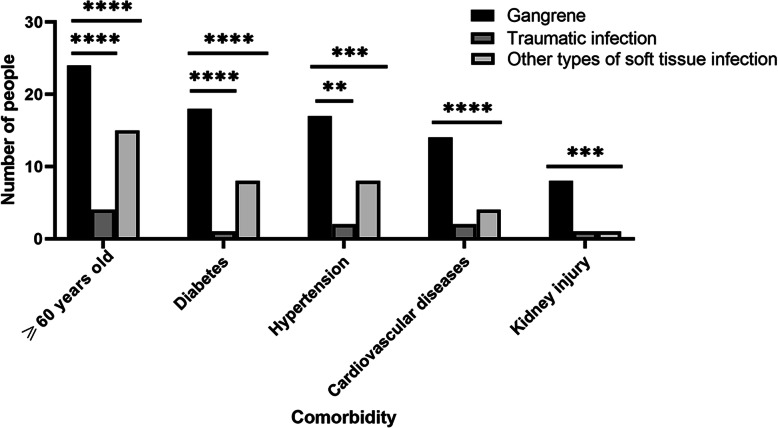
Difference of COVID-19 risk factors in patients with different wounds. *n* = 81, *p* value > 0.05 not marked, *< 0.05, **< 0.01, ***< 0.005, ****< 0.001s

As of May 20, 2020, 78 people (96%) had been discharged from the hospital with an average length of stay of 15.8 days (SD 14.2), compared to 3 patients who remained in the hospital with a more prolonged clinical course and a significantly longer length of stay of 39.7 days (SD 8.7) (*P* = 0.0054), as well as more complex wound and more complications (Fig. 4). The mean length of hospital stay was long for all patients, with varying lengths of stay, and the average timespan is 16.7 days (SD 14.7) (Table 1), while there was no significant difference in discharge time for patients with different types of wound (*P* = 0.40) (Fig. 5). According to the analysis of the risk factors of COVID-19 in patients with different wounds, gangrene accounted for the biggest number of patients with risk factors of the three types of chronic wounds, especially for the advanced age and diabetes, which were significantly different from the other two groups (*P* < 0.001) (Fig. 6).

## Discussion

The First Affiliated Hospital of Zhejiang University School of Medicine (Zhejiang University First Hospital for short), as a designated hospital for patients with severe novel coronavirus pneumonia in Zhejiang Province, admitted and treated a total of 105 confirmed cases (including 78 cases of severe and critical cases) and excluded 183 suspected cases, without any cases of COVID-19 among medical staff and patients in the hospital. Simultaneously, a medical team was sent to take over the new coronavirus pneumonia ward in several hospitals in Wuhan, and at the same time, completed the rescue and treatment tasks, the medical staff also achieved zero infection. The prevention and control of hospital-acquired infections have played a key role in the prevention and control of this COVID-19 epidemic, and we, therefore, hope to share our experience of diagnosis and treatment with other countries in order to combat this global epidemic.

SARS-CoV-2 is a newly discovered virus for which there is currently no effective specific vaccine for its infection; therefore, isolation of infected persons and prevention and control of at-risk populations is of great importance. Identification of COVID-19 in patients with chronic infective wound presenting with nonspecific symptoms is challenging and difficult, especially in non-outbreak areas, where early clinical and imaging presentation of COVID-19 was not characteristic [29]. At the same time, nearly 73% of patients infected with SARS-CoV-2 are men, as well as old age, diabetes, and lung disease are also independent risk factors; thereby, the control of such groups is also of particular importance [3, 30].

In a survey of chronic wound patients documented in China between 2018 and 2019, it was found that 76.74% of patients over 50 years of age had comorbid underlying diseases, of which 78.25% had comorbid underlying diseases, and the top four were diabetes, cardiovascular disease, hypertension, and respiratory disease [31, 32]. Our study also shows that patients with chronic infective wound are predominantly older men with multiple underlying conditions, who happen to be at high risk for coronavirus infection. In addition, chronic wound often requires specialized clinical care due to the susceptibility of the lesion and the prolonged changes in the disease; otherwise, their simple superficial infections will worsen, causing systemic infections, amputations, and even death [33]. In our study, as we reviewed patients’ medical records, we found that many patients were unable to seek medical attention in time during the epidemic, which resulted in delaying the best opportunity for treatment and causing the deterioration of the wound to require surgical treatment; therefore, it is of great importance to arouse people’s attention to wound care during the epidemic. Just for instance, an elderly male (66 years old) was stabbed with a brick in his right interosseous after a sudden fall. Without sufficient medical support, only a simple hemostatic bandage was performed, and 20 days later, the patient developed a severe infection in his right interosseous region along with significant hand swelling and osteofascial ventricular syndrome (Fig S1). Eventually, he underwent chronic ulcer repair and decompression of compartment hypertension, which will be described later. It can be seen that appropriate antibiotic therapy and timely surgical debridement or drainage are crucial for the control of the disease in these patients, whereas on the other hand, it leads to the inevitable need for regular hospital visits, which increases the risk of COVID-19 infection due to transportation and in-hospital treatment. Therefore, balancing the control of wound progression and the reduction of the risk of coronavirus infectious during COVID-19 epidemic is a particular challenge for chronic wound patients.

Based on the WHO recommendations for the clinical management of COVID-19, the Novel Coronavirus Pneumonia Protocol (7th Edition) issued in China, and our treatment experience in the First Affiliated Hospital, College of Medicine, Zhejiang University, for all patients with chronic wound, we recommend the application of APPs that used to personal health status identification [34], such as the implementation of the Green Health Code in China, which plays an important role in the identification of the epidemiological history of patients who require multiple trips to hospitals or other place, along with the full integration of telemedicine and nosocomial infection control during the COVID epidemic to reduce patient risk of infection [35]. First, patients are advised to seek medical help on the Internet; then, doctors learn the patient’s epidemiological history, symptomatic signs, and other information online; triage the patient; and, on the basis of stabilization, if possible, advise the patient to self-treatment, guided therapy, or nearest treatment, to reduce the risk of exposure during transportation [36]. When patients have to go to the hospital, the information obtained is used to classify patients into low-risk and middle-to-high-risk patients which applies to implement dual-channel triage, and meanwhile control the waiting spacing, and reconfirm the patient’s medical history and other information at the triage desk, to reduce the risk of infection in outpatients. Besides, the hospital must strengthen the education of infection-prevention knowledge for all personnel, even for cleaning personnel, and the storage of protective materials and indoor ventilation measures. In the wards, we suggest that hospitals with the conditions can set up a special branch to receive infected or suspected patients, such as our hospital has a special area of Zhijiang hospital; otherwise, emergency isolation wards should be set up within the ward for medium- to high-risk patients with COVID-19 and general wards. In addition, the wards can be divided into contaminated areas, buffer zones, and clean-up areas, and, if possible, medical equipment and responsible medical personnel can be fixed in each bed to achieve more precise protection. Chaperones in the ward should also avoid excesses, and educate the patients and their relatives on infection prevention and control. In the case of surgical patients [36–38], our recommendation is to avoid surgery during a pandemic as much as possible and, in the case of limited duration surgery, to avoid the risk of aerosol transmission of the virus by selecting a negative pressure operating room or at least an operating room with independent ventilation. Furthermore, avoid invasive airway manipulation as much as possible, e.g., nerve block anesthesia for anesthetic measures, thus reduce the risk of respiratory tract infection.

In the course of medical treatment, it is necessary not only to pay attention to contagion among patients, but also to be alert to medical personnel as a source of contagion, who is also the backbone of the battle against the epidemic; therefore, social isolation and personal protection of medical personnel should also be paid attention [35]. When physicians share a room with COVID-19 patients, their chances of infection are greatly increased, especially when performing invasive procedures, and then, they may become new source of infection to their intimates [39, 40]. A single surgical procedure can trigger transmission of the virus, and when a clinician is faced with a chronic trauma patient with COVID-19, multiple dressing changes inevitably increase the risk of COVID-19 transmission. Thus, doctors should not only take care to keep themselves at a distance from the patient’s airway and isolated from the patient’s body fluids, but should also practice social distance between their colleagues. Meanwhile, given the enormous medical stress caused by the COVID-19 epidemic, it is also important for health care personnel to choose appropriate ways to release stress to avoid serious mental illness [41].

Meanwhile in this study, we found that among three types of wounds, patients with other types of soft tissue infections were the most common; thus, these patients were the most easily encountered in clinical work during the COVID-19 epidemic. The following is gangrene due to vascular occlusion, which is also the type of chronic wound with the longest hospital stay and the most risk factors for COVID-19 as shown in Fig. 6. Not only that, but the two types of wound are also slightly similar in clinical presentation, and in our study, most patients presented with lesions of the extremities, with localized swelling, redness, fever of skin, and pain manifesting early in the disease. When it comes to COVID-19, it may perform the same [42, 43].

Therefore, in addition to the above-recommended management requirements for all chronic wound, we suggested that for these two kinds of patients we not only classify them according to the severity of their condition, but also strengthen our comprehension of the epidemiological history and underlying disease status of these patients and stratify the risk of COVID-19 infection [33, 44]. Through the implementation of Internet telemedicine and electronic follow-up, as mentioned above, for patients with mild and manageable disease, we would recommend them to take medication and isolate themselves at home, perform appropriate activities, and monitor changes in the lesion area. While for patients with thrombotic disease, the detection of INR index is very important, so we suggest that, based on a stable condition, the time interval between visits to the hospital for INR testing can be extended, or using home testing or staggered testing in hospital to reduce the risk of contact transmission and nosocomial COVID-19 infection. In the case of inpatients, after the management of the block by separating hospital district, ward, and sector area to reduce the risk of nosocomial infections, the choice of treatment options we recommend are that, on the basis of maintaining the stability of the disease, priority should be given to drug treatment; invasive treatment such as surgery or intervention need to be postponed as far as possible. If the underlying disease is more serious, the patient should first undergo a multidisciplinary consultation to control the comorbidities, and after the comorbidities have been stabilized, the patient can undergo wound treatment and should be well prepared for repeated treatment. For example, during this outbreak, we received a middle-aged male patient (47 years old) with hypertension, diabetes, and severe heart disease, including dilated cardiomyopathy, heart failure, and coronary heart disease, and was admitted to the hospital for soft tissue infection caused by a wood barb wound in his left hand (Fig S2). COVID-19 infection was ruled out after prehospital screening, and the patient was transferred to a general ward. After examining the patient’s cardiac function (40% left ventricular diastolic function, moderate stenosis of the left anterior descending branch of the coronary artery, and mild stenosis of the right coronary artery), we invited the cardiology department for a consultation to assess the risk of surgery. Then the patient underwent finger amputation and wound repair (Fig S3). Since the patients were generally poor which contribute to the unsatisfactory invasive healing, again, we performed 7 times of chronic ulcer clearing and repair procedures after adequate preoperative preparation and patient evaluation (Fig S4). Finally, the patient was well-covered by the graft wound and was discharged smoothly for home anti-infection, treatment of underlying diseases, and regular wound reviewing (Fig S5). For patients combined with COVID-19 infection, one difference is that attention needs to be paid to the interactions between COVID-19 clinical trial drugs and anti-infection, antiplatelet, or other drugs [45]. Meanwhile it is also important to prevent these diseases, which may increase the length of hospitalization and the risk of COVID-19 infection, and may make it more difficult for patients with COVID-19 infection to be treated clinically. We, therefore, recommend preoperative and intraoperative prophylactic antibiotic use for all surgical patients. And in the case of bedridden or limited mobility patients, pharmacological prophylaxis should be considered after individual assessment, given their increased risk of thrombosis.

When it comes to trauma-induced chronic wound, as it has an inevitable need for surgical treatment such as debridement, suturing, and drainage, we recommend that all such patients undergo screening for COVID-19 like chest CT and nucleic acid testing, and considering that such inpatients require multiple surgeries and are prone to fever during hospitalization, thus, a well-developed process is needed, such as timely review of chest-CT and invitations to the infection unit for consultations. For COVID-19-positive or high-risk patients, they should be immediately isolated and operated in the designated negative-pressure operating room, along with disposable surgical items as far as possible, and streamline the required medical staff. Post-operative patients continue to receive isolation therapy and associated trauma care as soon as they return to the ward. Patients who are COVID-19 negative or at low risk should also be seen in a clean area, and surgical masks should be worn to reduce the chance of droplet transmission during the surgery. At the same time, the surgical instruments should be strictly disinfected, packed, and transported, and the operators should be as few as possible and wear protective equipment. Strict intraoperative adherence to routine aseptic procedures, with thorough debridement and negative pressure suction, is also important for subsequent wound recovery. Postoperatively, antibiotics can be applied for infection prevention, and regular wound cleaning and replacement of negative pressure suction devices can be performed to promote wound recovery (Fig S6). After discharge, patients should be followed up electronically, including guidance on wound care and the use of antibiotics, and patients should be asked to change their medications regularly at their nearest clinic, so that they can receive efficient and adequate medical support during the epidemic.

There are still some limitations to this study. Firstly, this center is not the epicenter as Wuhan is; thus, novel coronavirus pneumonia has a smaller infection base in this region, and there is a lack of a richer patient population and a large enough sample size to support the results more reliably, and it is hoped that the findings here will encourage larger cohort studies in other regions or possibly some randomized controlled trials. Secondly, since this is a retrospective study, it is not possible to conduct a prospective study on the probability of COVID-19 infection in all patients with chronic wounds, and it is hoped that this will inspire other researchers to conduct studies on this issue and raise the awareness and treatment of this condition among clinicians and the public.

## Conclusions

In general, patients with chronic wound have many risk factors for COVID-19 infection, and they should pay more attention to personal protection and social distance than normal people during medical treatment and daily life to reduce exposure risk. When such patients present with fever, dry cough, worsening lesion, etc., clinicians should be alerted to the possibility of their infection and carry out the risk stratification of COVID-19, as well as the grade diagnosis and treatment based on the severity of the disease. Priority is given to non-invasive treatments such as medication and physiotherapy for hospitalized patients, along with the postponement of surgery as far as possible [37]. Once surgery starts, there need to be strict aseptic operation and personal protection for both patients and doctors. In patients with comorbidities, the comorbidities need to be kept stable, and multidisciplinary consultations should be carried out if necessary. Patients who have been infected with COVID-19 combined with chronic infective wound should be closely monitored and isolated for treatment, negative pressure wards should be used during surgery, and standardized preoperative, intraoperative, and postoperative treatment should be carried out to improve their prognosis.

## Supplementary information

**Additional file 1: Figure S1.** Lesion of a patient with incisional infection: (A) dorsal (B) palm, there was redness, swelling and pain in the right hand of the patient, along with high local skin temperature, fluctuating sensation on palpation at the swollen area, and a black, crusted brick scratch wound was visible at the large interfiscial area of the palm. The patient had a 30-year history of previous kidney transplantation and a 30-year history of gout, and a resuscitation history in our hospital for an acute heart attack before seven months.

**Additional file 2: Figure S2.** Preoperative hand appearance of the patient: (A)palm (B) lateral, the patient's left hand is purple-black in color, with poor local skin blood transport, severe swelling of the forearm and palm of the left hand with pressure pain.

**Additional file 3: Figure S3.** Manifestations of the patient's hand at the first operation: (A) large amount of purulent fluid and necrotic tissue visible after fascial ventricular decompression, (B) palmar surface of the patient after finger amputation, (C) back of the patient after finger amputation.

**Additional file 4: Figure S4.** Poor wound healing in postoperative patients. 7 days postoperatively (A) palmar (B) back. 27 days postoperatively (C) palmar (D) back. Postoperatively 47 days (E) palmar (F) back.

**Additional file 5: Figure S5.** Patient wound appearance at discharge (57 days postoperatively) (A) palmar (B) back.

**Additional file 6: Figure S6.** Changes of wound surface in patients with incision infection. Preoperative hand condition: (A) back (B) palmar, same patient as Figure 7 introduced. The condition of the right hand during the operation: (C) the patients underwent compartment incision and decompression, and a large amount of purulent fluid was found during the operation. (D) A large number of necrotic tissues were found and had be removed during the operation. The condition of the right hand after operation: (E) at the time of ulcer repair. (F) VSD was removed on the 3rd, 8th, 13th and 16th day after operation. Fresh granulation tissue and extensor tendon were found on the back of hand.

## Data Availability

The dataset supporting the conclusions of this article is included with the article.
