# Supplementary figures and images for: Exposure risk of patients with chronic infectious wounds during the COVID-19 outbreak and its countermeasures
Source: J Orthop Surg Res. 2020 Oct 2;15:452. doi: 10.1186/s13018-020-01976-0 (PMC7530861; doi:10.1186/s13018-020-01976-0)

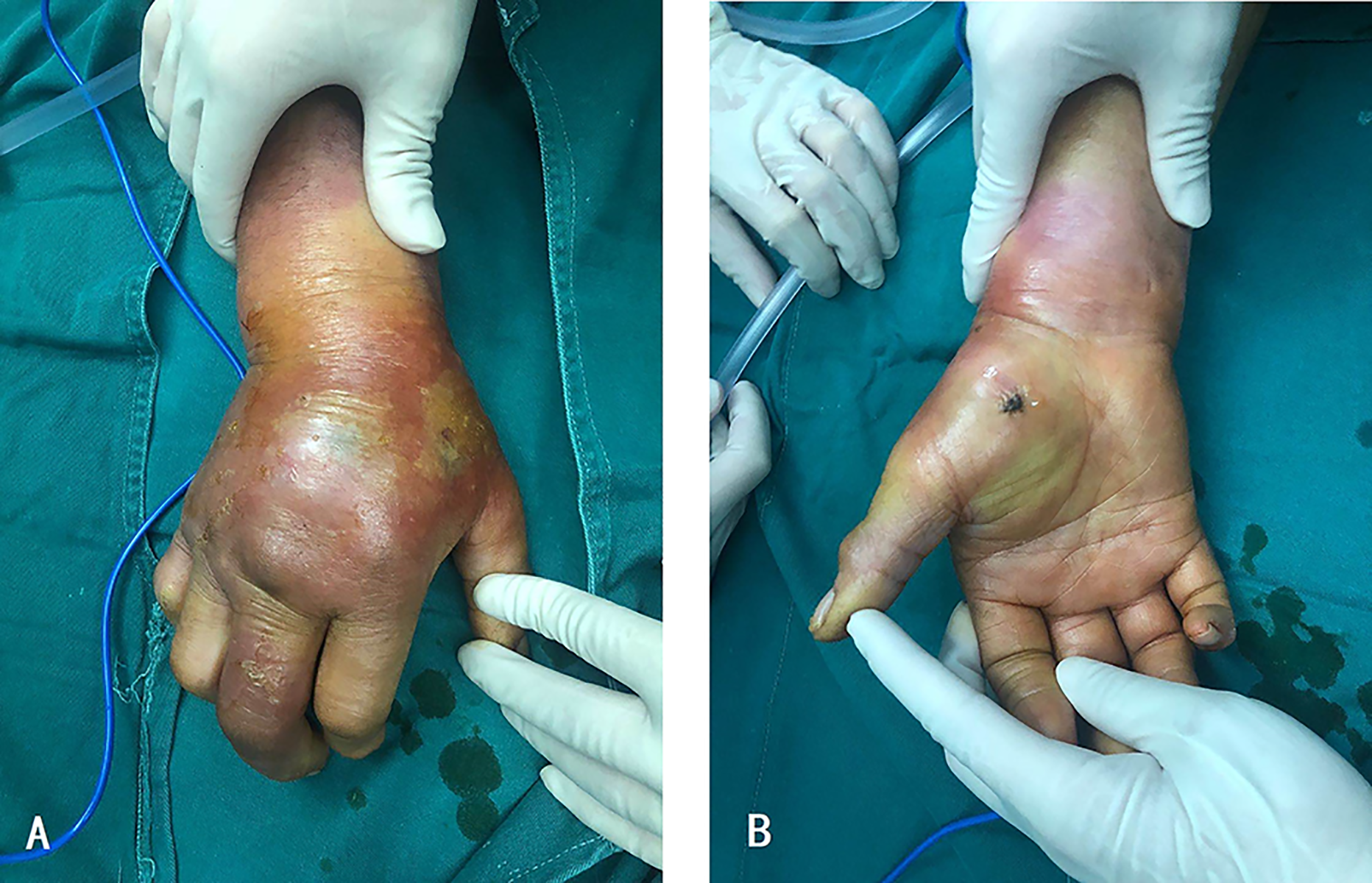

Supplement: Supplementary file 1 — Additional file 1: Figure S1. Lesion of a patient with incisional infection: (A) dorsal (B) palm, there was redness, swelling and pain in the right hand of the patient, along with high local skin temperature, fluctuating sensation on palpation at the swollen area, and a black, crusted brick scratch wound was visible at the large interfiscial area of the palm. The patient had a 30-year history of previous kidney transplantation and a 30-year history of gout, and a resuscitation history in our hospital for an acute heart attack before seven months. [file 13018_2020_1976_MOESM1_ESM.tif]

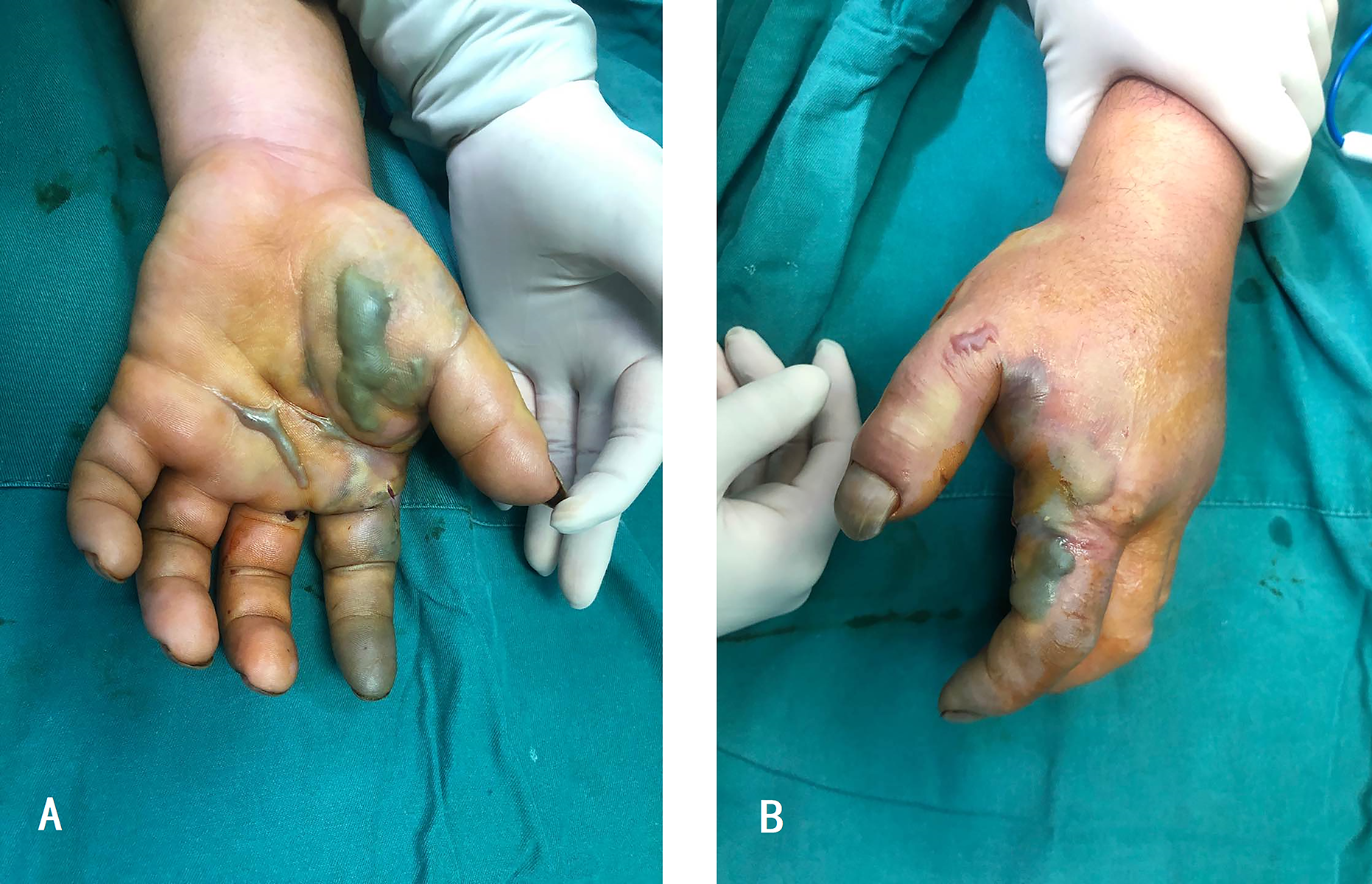

Supplement: Supplementary file 2 — Additional file 2: Figure S2. Preoperative hand appearance of the patient: (A)palm (B) lateral, the patient's left hand is purple-black in color, with poor local skin blood transport, severe swelling of the forearm and palm of the left hand with pressure pain. [file 13018_2020_1976_MOESM2_ESM.tif]

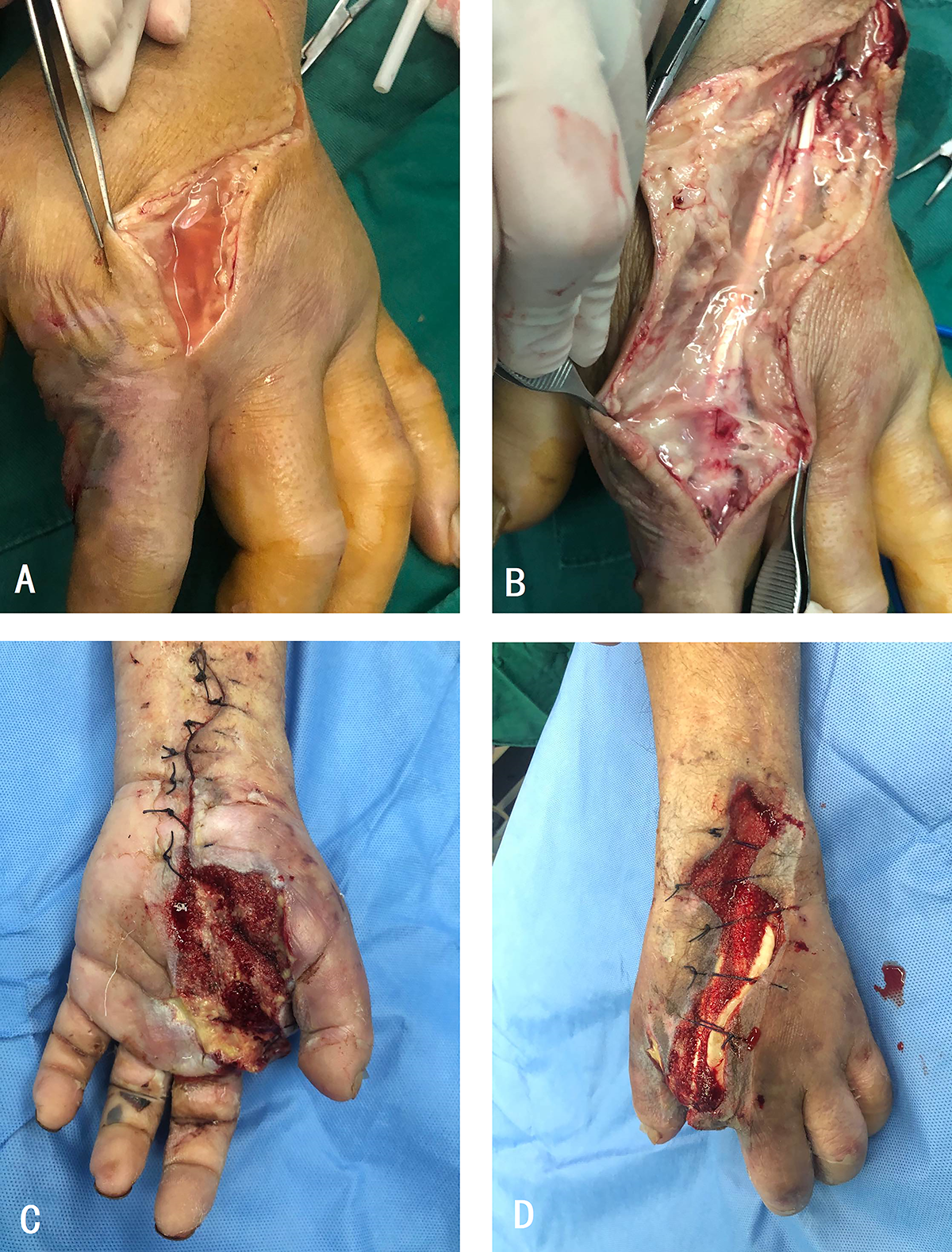

Supplement: Supplementary file 3 — Additional file 3: Figure S3. Manifestations of the patient's hand at the first operation: (A) large amount of purulent fluid and necrotic tissue visible after fascial ventricular decompression, (B) palmar surface of the patient after finger amputation, (C) back of the patient after finger amputation. [file 13018_2020_1976_MOESM3_ESM.tif]

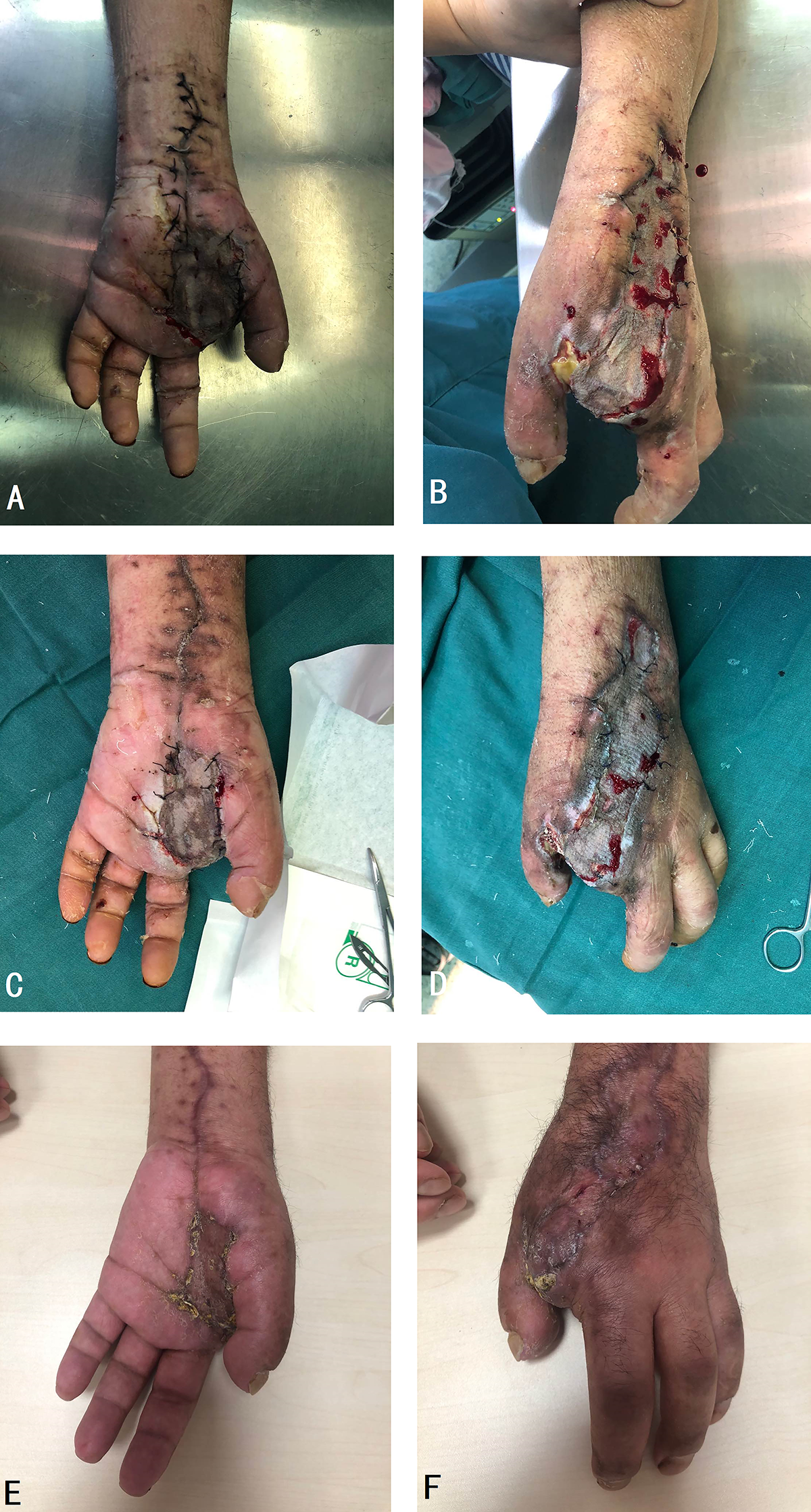

Supplement: Supplementary file 4 — Additional file 4: Figure S4. Poor wound healing in postoperative patients. 7 days postoperatively (A) palmar (B) back. 27 days postoperatively (C) palmar (D) back. Postoperatively 47 days (E) palmar (F) back. [file 13018_2020_1976_MOESM4_ESM.tif]

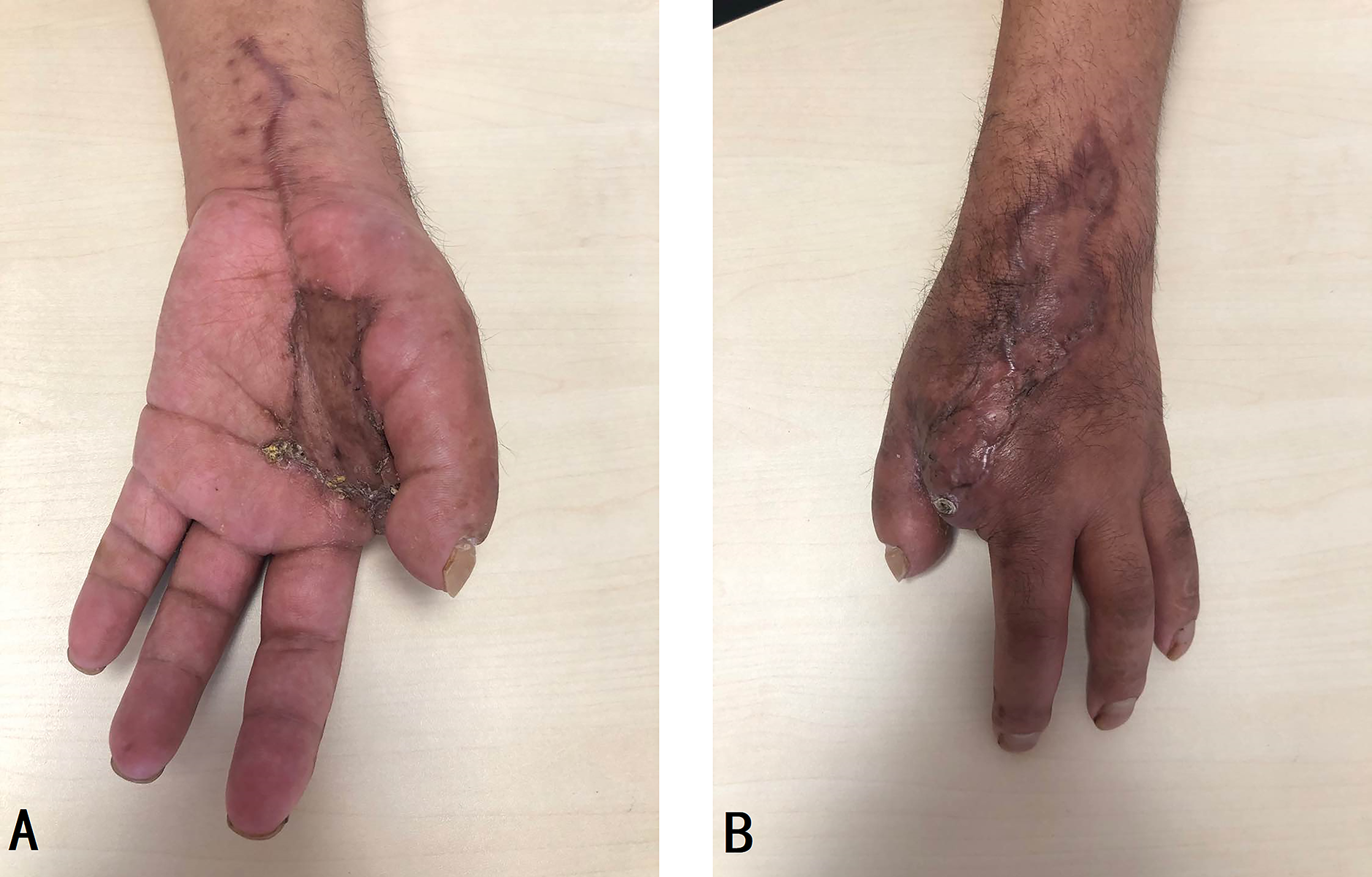

Supplement: Supplementary file 5 — Additional file 5: Figure S5. Patient wound appearance at discharge (57 days postoperatively) (A) palmar (B) back. [file 13018_2020_1976_MOESM5_ESM.tif]

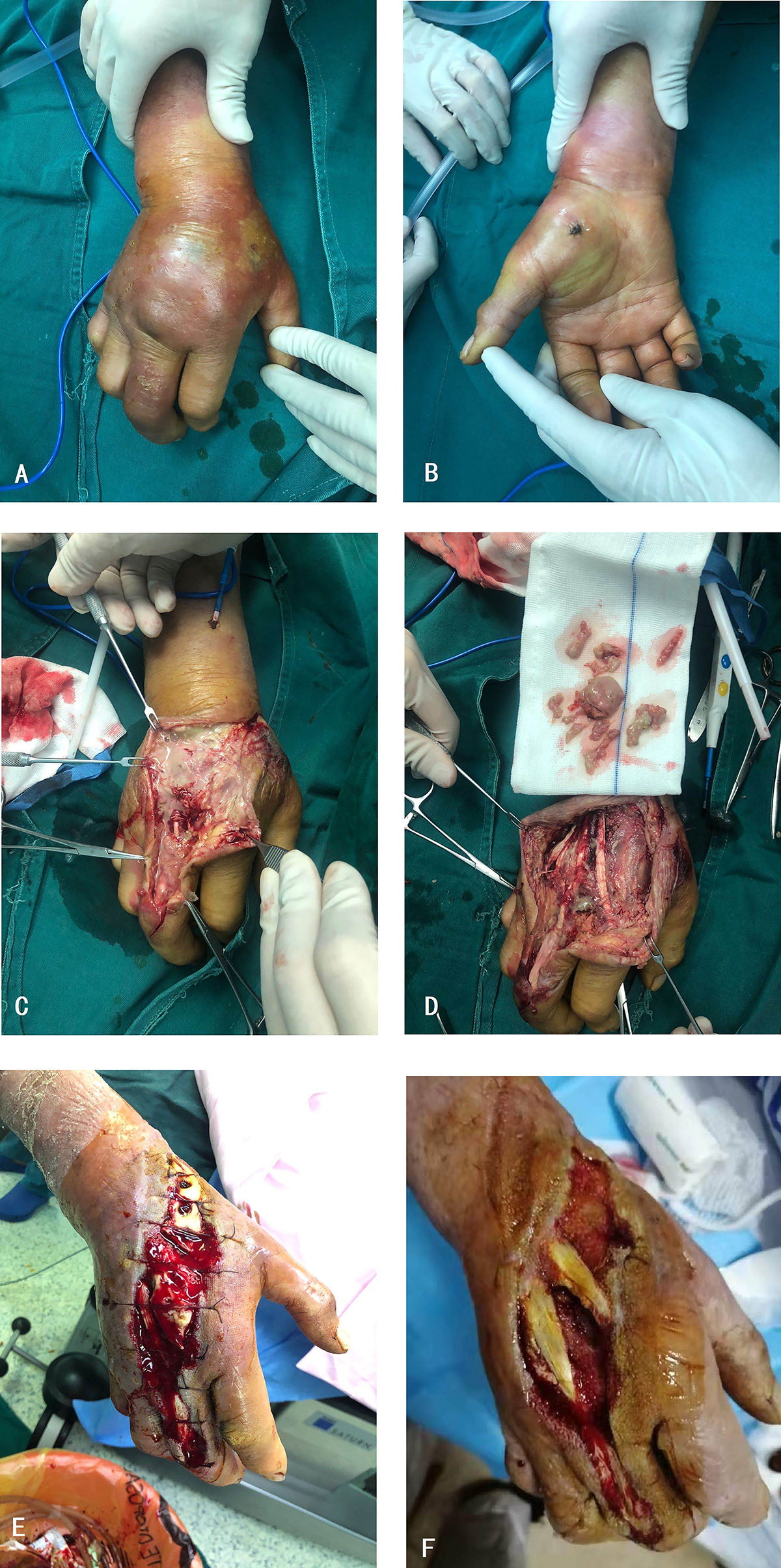

Supplement: Supplementary file 6 — Additional file 6: Figure S6. Changes of wound surface in patients with incision infection. Preoperative hand condition: (A) back (B) palmar, same patient as Figure 7 introduced. The condition of the right hand during the operation: (C) the patients underwent compartment incision and decompression, and a large amount of purulent fluid was found during the operation. (D) A large number of necrotic tissues were found and had be removed during the operation. The condition of the right hand after operation: (E) at the time of ulcer repair. (F) VSD was removed on the 3rd, 8th, 13th and 16th day after operation. Fresh granulation tissue and extensor tendon were found on the back of hand. [file 13018_2020_1976_MOESM6_ESM.tif]
